# Preventing Colitis-Associated Colon Cancer With Antioxidants: A Systematic Review

**DOI:** 10.1016/j.jcmgh.2020.12.013

**Published:** 2021-01-05

**Authors:** Thergiory Irrazabal, Bhupesh K. Thakur, Kenneth Croitoru, Alberto Martin

**Affiliations:** 1 Department of Medicine, University of Toronto, Toronto, Ontario, Canada; 2 Department of Immunology, University of Toronto, Toronto, Ontario, Canada; 3 Department of Medicine, Division of Gastroenterology, Mount Sinai Hospital, Toronto, Ontario, Canada

**Keywords:** Colitis, Inflammatory Bowel Disease, Colorectal Cancer, Antioxidants, DNA Damage, AOM, azoxymethane, AT1, angiotensin II type 1, CAC, colitis-associated colon cancer, CAT, catalase, CRC, colorectal cancer, DSS, dextran sodium sulfate, DUOX2, dual oxidase 2, Gpx, glutathione peroxidase, GST, glutathione-S-transferase, GSTTT1, glutathione-S-transferase theta 1, HFD, high-fat diet, H_2_O_2_, hydrogen peroxidase, IBD, inflammatory bowel disease, MMR, mismatch repair, mtROS, mitochondrial ROS, NAC, N-acetylcysteine, NOX, nicotinamide adenine dinucleotide phosphate oxidase, O2^•-^, superoxide, PRDX, peroxiredoxin, RNI, reactive nitrogen intermediaries, ROS, reactive oxygen species, SOD, superoxide dismutase, UC, ulcerative colitis, vitC, vitamin C, vitE, vitamin E, 8-oxoG, 8-oxo-7,8-dihydro-2′-deoxyguanosine

## Abstract

Inflammatory bowel disease (IBD) patients have an increased risk of developing colitis-associated colon cancer (CAC); however, the basis for inflammation-induced genetic damage requisite for neoplasia is unclear. Several studies have shown that IBD patients have signs of increased oxidative damage, which could be a result of genetic and environmental factors such as an excess in oxidant molecules released during chronic inflammation, mitochondrial dysfunction, a failure in antioxidant capacity, or oxidant promoting diets. It has been suggested that chronic oxidative environment in the intestine leads to the DNA lesions that precipitate colon carcinogenesis in IBD patients. Indeed, several preclinical and clinical studies show that different endogenous and exogenous antioxidant molecules are effective at reducing oxidation in the intestine. However, most clinical studies have focused on the short-term effects of antioxidants in IBD patients but not in CAC. This review article examines the role of oxidative DNA damage as a possible precipitating event in CAC in the context of chronic intestinal inflammation and the potential role of exogenous antioxidants to prevent these cancers.

**Summary**

Preclinical studies suggest a role for oxidative molecules in the pathophysiology of colitis-associated cancer. This review analyzes evidence for DNA oxidation as a precipitating event in gastrointestinal cancers and synthesizes an argument for the use of antioxidants as a viable therapeutic treatment to prevent colitis-associated colon cancers.

Inflammatory bowel disease (IBD) is a multifactorial chronic inflammatory disorder associated with dysregulation in the interaction between the host’s immune system and the environment within the gastrointestinal tract. Chronic inflammation of the intestinal epithelium is positively associated with cancer development, and although there are several mechanisms by which inflammation could induce epithelial damage, only a few of those point to a direct source of the DNA lesions necessary for cellular transformation and cancer initiation. The oxidant environment created by activated inflammatory cells in the intestinal epithelium has been associated with carcinogenesis.^1^, ^2^, ^3^, ^4^ Although linked to cancer initiation, reactive oxygen species (ROS) also act as signalling molecules that regulate multiple signaling pathways associated with mitogenesis, immune and stress response, and autophagy and are therefore required to maintain homeostasis.^5^ In this review, we will discuss the evidence that supports the notion that chronic intestinal oxidation is one of the main factors leading to DNA lesions that promote carcinogenesis in IBD patients (Figure 1). This review will also discuss how antioxidants could be used to suppress tumor development within the inflamed intestinal tissue.

**Figure 1:**
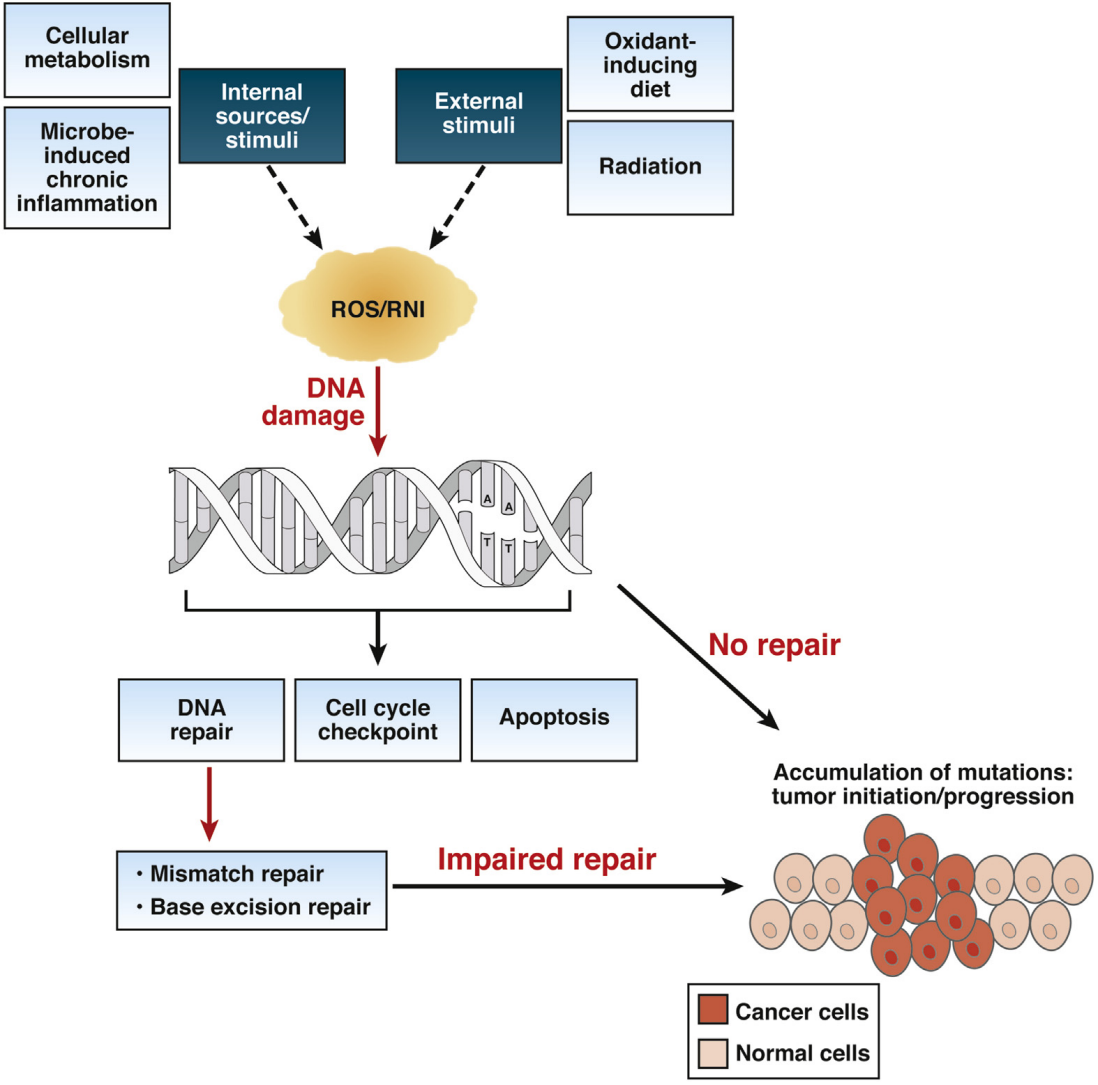
**Unrepaired oxidative DNA damage can lead to cancer-promoting accumulation of mutations.** Several stimuli from both endogenous and environmental sources induce increased production of ROS and RNI that can directly produce mutagenic DNA lesions. Oxidative DNA damage is primarily repaired by MMR repair and base excision repair pathways involving the excision of modified bases followed by repairing of the gaps. Oxidative DNA damage could exceed the repair capacity of these DNA repair pathways, or deficiency in these specific DNA repair pathways can lead to the accumulation of oncogenic mutations, precipitating cellular transformation and ultimately tumor initiation and/or progression.

## What Is the Source of Oxidative Molecules and DNA Lesions in the Intestinal Epithelium?

There are several sources of oxidative molecules in the intestinal epithelium. Classically activated macrophages, infiltrating neutrophils, and intestinal epithelial cells are all equipped with enzymes that produce ROS and reactive nitrogen intermediaries (RNI) in response to the gut microbiota, specific gut pathogens, or other stimuli. ROS and RNI are normally produced to keep microbes in line and maintain homeostasis within the intestine. For example, the nicotinamide adenine dinucleotide phosphate oxidase (NOX) 2, which produces superoxide (O_2_^•-^), is expressed in macrophages, dendritic cells, and neutrophils that infiltrate the lamina propria.^6^ Intestinal epithelial cells express the O_2_^•-^ producing enzyme NOX1 and the hydrogen peroxide (H_2_O_2_) producing enzyme dual oxidase 2 (DUOX2). Usually O_2_^•-^ is further converted into the more stable molecule H_2_O_2_ by the enzyme superoxide dismutase (SOD). In phagocytes, H_2_O_2_ is further transformed into hypochlorous acid, which has antimicrobial properties. In addition, macrophages, epithelial cells, and neutrophils also express the enzyme inducible nitric oxide synthase, which produces the highly diffusible molecule NO^•^.^6^^,^^7^

Although ROS is necessary for various cellular functions,^8^, ^9^, ^10^, ^11^, ^12^ excessive accumulation causes damage to biological molecules. Consequently, tissues are armed with several defense mechanisms including an intricate antioxidant defense system. The antioxidant defense system primarily functions through (1) limiting the excessive production of ROS/RNI, (2) scavenging free radicals, and (3) converting toxic free radicals into less toxic molecules.^13^ An incompetent or dysregulated antioxidant system is associated with inflammatory diseases. Accordingly, mice deficient in nuclear factor erythroid-2–related factor 2, a master regulator of antioxidant responses in tissue through transcriptional regulation of antioxidant genes, develop colitis and colitis-associated cancer (CAC).^14^

During chronic inflammation, ROS and RNI surpass the physiological antioxidant detoxifying capacity of cells, leading to the generation of high amounts of oxidant molecules. H_2_O_2_ can react with transition metals via Fenton reaction and produce the highly reactive hydroxyl radical (HO^•^).^7^ On the other hand, ^•^NO can react with O_2_^•-^ to generate the highly reactive molecule peroxynitrite (ONOO^-^).^15^ When these oxidant molecules are not neutralized by intracellular antioxidants, these agents induce cell membrane damage and cancer-causing DNA lesions.^1^^,^^3^^,^^16^ In addition, inflamed colons in IBD patients and mice with colitis have decreased expression of antioxidant enzymes such as glutathione-S-transferase theta 1 (GSTT1). GSTT1 is not only necessary for the detoxification of oxidative free radicals, but it is also necessary to induce goblet cell differentiation and mucin production in response to triggers such as interleukin 22 and H_2_O_2_.^17^ Because mucus produced by goblet cells is the first barrier that protects intestinal cells from the microbiome, this study suggests that GSTT1 deficiency would disrupt the epithelial barrier, generating a positive loop for oxidative epithelial damage.^17^

## Inflammation, Diets, and Oxidative Environment

Several innate immune cells produce O_2_^•-^, H_2_O_2_, and NO as a defense mechanism. However, some studies suggest that an increase in oxidative molecules can precede inflammation. For example, an inhibitor of the DNA repair enzyme OGG1 (8-oxoguanine DNA glycosylase 1) has been shown to prevent proinflammatory gene expression and cell recruitment in mouse lungs,^18^ suggesting that repair of oxidative DNA damage induces inflammation. Other studies showed that mitochondrial ROS (mtROS), generated when electrons leak from Complex I and III during oxidative phosphorylation and react with oxygen to form O_2_^•-^, can act as a signal-transducing molecule that either activates the NLRP3 inflammasome inducing the production of proinflammatory cytokines^19^^,^^20^ or mediates an increased mitogen-activated protein kinase signaling that induces inflammatory cytokine production after TLR4 activation.^21^ Furthermore, gene transfer of the mitochondrial antioxidant enzyme GSTT1 into the colon of mice confers protection against colitis,^17^ suggesting that in some cases, an increase in mtROS could precede chronic intestinal inflammation. However, mtROS can have anti-inflammatory properties as well because it can protect the intestine from inflammation by inducing polarization of alternatively activated macrophages and a reduction in the production of proinflammatory cytokines.^22^ Overall, these data suggest that mtROS must surpass a physiological threshold to lead to activation of inflammatory pathways in the intestine.

Another factor that can induce an oxidative environment that results in intestinal permeability and inflammation are high-fat diets (HFDs).^23^^,^^24^ Non-esterified long chain saturated fatty acids present in HFD can increase expression of *Nos2* and endoplasmic reticulum stress in goblet cells, which in turn triggers a reduction in the production of the mucus barrier and creates a positive feedback loop for inflammation.^23^ In addition, mice fed a HFD had decreased expression of tight junctions proteins and MUC2 and increased expression of the enzymes NOX1, NOX4, and NOS2, which produce O_2_^•-^, H_2_O_2,_ and NO, respectively.^24^ However, none of these molecules or their oxidative effects were directly measured. Although the mechanism by which HFD induces the expression of these ROS-producing enzymes is not clear, it is possible that intestinal permeability associated with deficiencies in tight junctions and/or reduced mucus layer lead to the penetration of molecules such as lipopolysaccharide through the epithelial layer of the gastrointestinal tract. This in turn can lead to the induction of ROS-producing enzymes in immune cells. Interestingly, flavonoid anthocyanins could revert HFD-induced intestinal permeabilization and endotoxemia in part by modulating NOX expression and preventing the production of RNI.^24^ This finding supports the idea that HFD-induced oxidative environment can lead to a positive feedback loop for intestinal permeability and intestinal oxidation. Anthocyanins also showed a promising anti-inflammatory potential in a small trial in ulcerative colitis (UC) patients,^21^^,^^25^ and because HFD is a risk factor for IBD,^26^ it is possible that anthocyanins could counteract the initial ROS-related processes that precede chronic intestinal inflammation.

Overall, it seems that the source of oxidative damage in the intestine could be dependent or independent of inflammatory cells. Mitochondrial dysfunction and certain diets can mediate an increase in the production of O_2_^•-^ and NO in the intestinal epithelium,^20^^,^^21^^,^^23^, ^24^, ^25^^,^^27^ which in turn can activate inflammatory pathways and may lead to mutations that perpetuate inflammation and/or initiate cancer. However, in the majority of cases, it is likely that inflammation is itself caused by a response to microbial stimuli that causes an oxidative environment that can precipitate cancer.

## How Is Oxidative DNA Damage Repaired?

HO^•^ and ONOO^-^ directly damage DNA via strand breakage or nucleotide oxidation. Guanine is the nucleotide with the highest oxidation potential,^15^ and oxidized guanine is commonly used to detect oxidative DNA damage. Elevated levels of oxidized guanine indicate that the oxidized environment has superseded the capacity of the cell to repair lesions caused by oxidation and therefore indicates potential oxidative damage.

**Figure 2:**
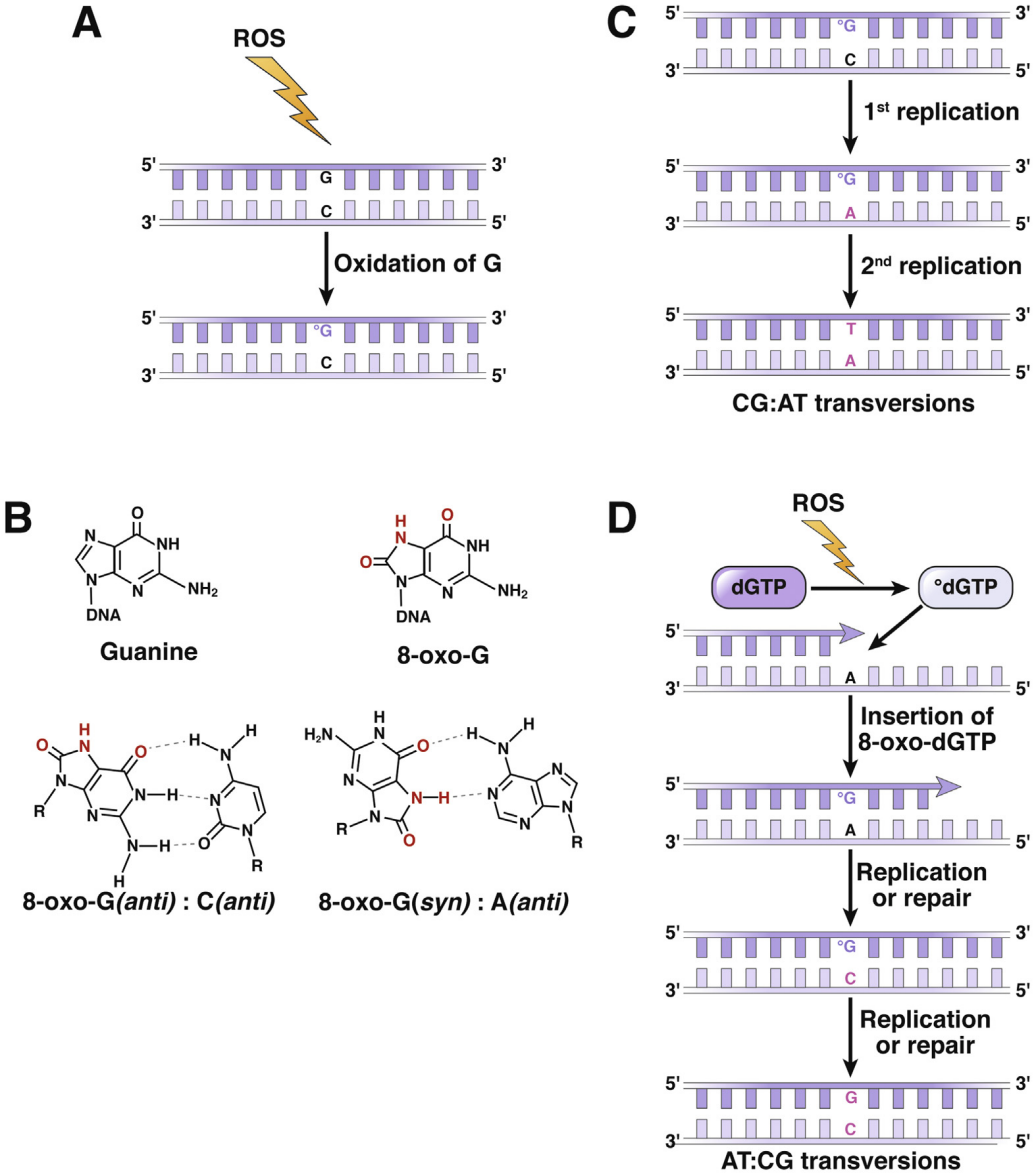
**Mutations caused by 8-oxoG.** (*A*) ROS-mediated oxidation of guanine (G) generates C:8-oxoG base pairs that are normally repaired by OGG1-initiated base excision repair. (*B*) 8-oxoG has base-pairing properties similar to thymine (T); therefore 8-oxoG in DNA during S-phase of the cell cycle leads to preferential insertion of adenine (A) opposite the 8-oxoG instead of cytosine (C) by replicative DNA polymerases. (*C*) A:8-oxoG mispairs can be recognized and repaired by MMR. However, if this mismatch is left unrepaired, a second round of replication will lead to C:G→A:T transversion mutation in one daughter cell. (*D*) Deoxyguanosine triphosphate (dGTP) in the nucleotide pool can be oxidized and incorporated into the nascent DNA strand opposite an A during replication, which can be repaired by MMR. However, A:8-oxo-G mispairs also can be processed through inappropriate MUTYH-initiated base excision repair, leading to the formation of C:8-oxo-G pairs, which could be further repaired by OGG1, generating an A:T→C:G transversion mutation. To avoid this, it is believed that cells avoid MUTYH activity during replication, giving preference to the MMR system. Therefore, lack of MMR activity is particularly an issue for highly proliferative tissues under oxidative environment such as gastrointestinal tract.

Guanine oxidation leads to the formation of 8-hydroxy-2′-deoyguanosine or 8-oxo-7,8-dihydro-2′-deoxyguanosine (8-oxoG), which can lead to mutations if not repaired (Figure 2). If 8-oxoG is not removed from the DNA before replication and because 8-oxoG preferentially pairs with an adenine, unrepaired 8-oxoG will ultimately lead to G → T and C → A transversion mutations (Figure 2). Multiple DNA repair systems repair oxidative DNA lesions at different stages of the cell cycle.^28^, ^29^, ^30^, ^31^ C:8-oxoG pairs in DNA are recognized and repaired by the base excision repair and the nucleotide excision repair systems.^29^^,^^31^ In addition, 8-oxoG nucleotides from the dNTP pool are usually removed by MTH1, and failure to do so can lead to incorporation of this oxidized base into the nascent DNA strand during DNA replication (Figure 2*D*).^29^, ^30^, ^31^ The mismatch repair (MMR) system has an especially important role in the repair of 8-oxoG lesions in highly proliferative tissues such as the intestinal epithelium because MMR-deficient human and mouse colonic tissue have exceptionally high levels of this DNA lesion.^3^

In the next sections, we discuss the role of endogenous and exogenous antioxidants on IBD and how these agents might prevent cellular transformation in inflamed intestinal tissue.

## Examining the Roles of Endogenous Antioxidants in IBD and CAC

The endogenous antioxidant defense system comprises both enzymatic antioxidants such as SOD, glutathione peroxidase (GPx), catalase (CAT), peroxiredoxin (PRDX), and thioredoxin as well as nonenzymatic antioxidants such as glutathione, alpha-lipoic acid, uric acid, melatonin, bilirubin, and ferritin. Multiple studies have found altered expression and/or activities of these antioxidant proteins in IBD and CAC patients, which suggest a role in disease pathology. SOD is a metalloenzyme that catalyzes the reduction of O_2_^•-^ to H_2_O_2_ and O_2_, whereas CAT catalyzes the detoxification of H_2_O_2_ to O_2_ and H_2_O. Patients with active Crohn’s disease have increased SOD activity, which returned to control levels at remission.^32^ However, CAT activity remained permanently inhibited and was independent of disease activity.^32^ In a different study, a reduction in CAT or total SOD activity was found to be associated with increased risk of colorectal cancer (CRC) and gastric adenocarcinoma, respectively.^33^

GPx, PRDX, and thioredoxin, the thiol-dependent proteins that catalyze the reduction of H_2_O_2_, lipid peroxides, and peroxynitrite, are found to be up-regulated in colonic mucosa of IBD and CRC patients compared with healthy subjects.^34^, ^35^, ^36^, ^37^, ^38^, ^39^, ^40^ The Gpx isoforms Gpx1 to Gpx4 are expressed in healthy gastrointestinal mucosa; however, their deficiency or genetic variants that affect their functions have been associated with increased mucosal damage and risk of developing CRC.^39^, ^40^, ^41^ Mice deficient in Gpx1, Gpx2, or Gpx3 develop normally but are susceptible to develop IBD and CAC upon *Salmonella* infection or azoxymethane (AOM)/dextran sodium sulfate (DSS) treatment.^42^^,^^43^ Compartmentalized expression of Gpx1 and Gpx2 is reported along the crypt-villus axis in the intestine; Gpx1 expression is predominantly found in the villus, whereas Gpx2 is localized mainly in the crypt.^44^ Gpx2 knockout mice have increased apoptosis and mitosis of crypt cells under selenium restriction. However, upon selenium supplementation in Gpx2^–/–^ mice, Gpx1 expression in the crypt increases, partially compensating for Gpx2 loss of expression and protecting from crypt cell apoptosis. This result suggests an overlapping complementary role of Gpx1 and Gpx2 in maintaining intestinal homeostasis.^44^ Accordingly, Gpx1 and Gpx2 double-knockout mice develop spontaneous colitis, dependent on excessive production of ROS by NOX1 and DUOX2.^45^, ^46^, ^47^

PRDXs are highly reactive peroxidases that account for the reduction of more than 90% of total cellular peroxides, while also crucial for maintaining physiological levels of cellular peroxides for vital cellular functions.^48^ All mammalian PRDXs, PRDX1–6, are overexpressed in the mucosa of active colitis and CRC patients, and their level in mucosa is positively correlated with disease severity and cancer metastasis.^34^, ^35^, ^36^, ^37^^,^^49^^,^^50^ Increased expression of PRDXs in diseased mucosa seems to be a host antioxidant defense response because PRDX4^–/–^ mice have higher disease severity and endoplasmic reticulum stress after DSS treatment.^51^ Studies suggest a dual role of PRDXs in cancer. PRDXs can either inhibit ROS-induced DNA damage and carcinogenesis or potentiate cancer progression through inhibition of ROS-mediated cell death in cancerous tissues.^49^^,^^50^ However, their role has not been investigated in CAC.

Researchers have examined nonenzymatic endogenous antioxidants and their roles in IBD and CAC. Some of these antioxidants such as glutathione, bilirubin, and uric acid are produced during normal metabolism, whereas melatonin is a hormone that is secreted from enterochromaffin cells in the intestine. Glutathione, the most important intracellular nonenzymatic antioxidant, is the substrate for glutathione-S-transferase (GST) that catalyzes the step of reduced glutathione conjugation with reactive electrophiles in the reduction of peroxides by glutathione peroxidase. The cellular level of glutathione was found to be reduced in intestinal mucosa of IBD patients, whereas reduced mucosal expression and/or activity of GST was also observed in IBD and CAC patients.^52^^,^^53^ Moreover, the serum levels of bilirubin, uric acid, and melatonin are found to be negatively associated with disease severity in IBD patients.^54^, ^55^, ^56^

The above findings not only imply the pathophysiological role of these endogenous antioxidants in IBD and CAC development but also suggest that the altered regulation of their expression in a diseased state is a compensatory response of the host to limit disease progression and severity.

## Testing Endogenous Antioxidants in IBD and CAC

Because epidemiologic studies have recognized the association of endogenous antioxidants with IBD and CAC pathophysiology, investigators have tested their therapeutic potential in these diseases. However, the short life span of recombinant enzymatic antioxidants in the gastrointestinal tract remains a barrier for therapeutic evaluation in intestinal diseases. Accordingly, attempts have been made to produce either stable proteins using genetic engineering or transgenic probiotic strains. Supplementation of genetically engineered *Lactobacillus fermentum* expressing recombinant SOD, hyperthermostable SOD from *Thermus thermophilus* HB27, or SOD mimics having enhanced stability and activity and ameliorated colitis severity in both mouse and human models.^57^, ^58^, ^59^, ^60^ In addition, treatment with genetically engineered *Lactobacillus casei* BL23 or *Streptococcus thermophilus* CRL807 expressing recombinant CAT or SOD restored endogenous antioxidant pools and reduced disease severity in colitis models.^61^^,^^62^ Remarkably, Ishihara et al^57^ observed a bell-shaped dose-response of SOD in colitis, demonstrating that a protective effect of SOD at lower doses is through reduction in colonic ROS level and ineffectiveness at higher doses is due to accumulation of H_2_O_2_. Accordingly, simultaneous administration of CAT restored the protective effect at higher doses of SOD.^57^

Recently, multiple studies demonstrate that boosting colonic H_2_O_2_ with probiotics can improve mucosal barrier integrity, increase colonization resistance, and suppress inflammatory responses in the colon, whereas exceeding the physiological levels of H_2_O_2_ could be detrimental.^10^, ^11^, ^12^ Because SOD converts O_2_^•-^ to H_2_O_2_ and physiological levels of H_2_O_2_ are important for gastrointestinal health, it is likely that H_2_O_2_ mediates the protective effect of SOD at lower doses in IBD.

Transgenic overexpression of another enzymatic antioxidant thioredoxin in mice led to reduced levels of tumor necrosis factor-α and interferon-γ upon DSS treatment compared with controls, suggesting an anti-inflammatory action of this enzyme.^63^ Accordingly, administration of recombinant human thioredoxin significantly ameliorated DSS-induced colitis and colonic inflammation in interleukin 10 KO mice.^63^ Thioredoxin can modulate the DNA binding properties of multiple transcriptional factors to regulate expression of inflammatory mediators.^63^^,^^64^ Overall, these studies report that restoring mucosal enzymatic antioxidants could be protective in IBD.

Nonenzymatic antioxidants as a therapeutic intervention in IBD have also been evaluated. Because IBD patients are depleted of glutathione in their gastrointestinal tracts, Ardite et al^65^ found that treating colitis-induced mice with glutathione attenuated acute colitis. In addition, ectopic expression of GSTTT1 1 in DSS-treated mice attenuated colitis severity via interleukin 22–dependent restoration of epithelial cell functions.^17^ Similar to glutathione, supplementing another thiol-containing endogenous antioxidant alpha-lipoic acid also reduced colitis and ileitis in animal models.^66^^,^^67^ Melatonin is another nonenzymatic compound recognized to have enteroprotective activity through its antioxidant and anti-inflammatory action.^68^ Exogenous administration of melatonin in experimental colitis models improved the disease pathology by reducing inflammation and epithelial damage.^69^, ^70^, ^71^ Melatonin also reduced the levels of oxidative DNA damage in colonic mucosa of IBD patients^72^; however, the effects on CAC were not evaluated.

Collectively, these studies suggest the therapeutic potential of endogenous antioxidants in IBD. However, few have evaluated the role of these agents in CAC in both preclinical models and in the clinic.

## Exogenous Antioxidants as Therapy in IBD and CAC

The current pathophysiological understanding of chronic inflammatory diseases and their association with endogenous antioxidants has encouraged researchers to develop therapeutics for IBD and CAC by using exogenous antioxidants. Exogenous antioxidants are substances that our body cannot produce and therefore must be provided as supplements from natural or synthetic sources. Synthetic antioxidants include compounds with antioxidant activities, precursors or mimics of endogenous antioxidants, and derivatives of amino acids such as propionyl-L-carnitine. Natural antioxidants consist of vitamins, polyphenolic compounds, polyunsaturated fatty acids, and trace metals. Numerous exogenous antioxidants have been investigated for their therapeutic potential in IBD, CAC, and CRC with promising results in preclinical models. However, most of the clinical trials do not validate the preclinical findings. Antioxidant supplementation was generally found to be ineffective or detrimental for cancer in most of the clinical studies^73^, ^74^, ^75^ (Tables 1 and 2), although some of the compounds used as antioxidants in these clinical studies do not have strict ROS-specific effects. Excessive ROS promotes mutagenesis through oxidative DNA damage and can trigger cancer development. However, it also has inhibitory roles on cancer progression through oxidation-induced cytotoxicity in cancer cells. Cytoplasmic ROS levels are significantly higher in cancer cells because of their increased metabolic activity compared with normal cells.^76^^,^^77^ To cope with oxidative damage-induced cytotoxicity, cancer cells depend on various mechanisms including an increase in their antioxidant pool for their survival. Thus, the concept of a negative correlation between antioxidant levels and cancer initiation/progression is now not universally valid,^77^ and elevating oxidation in tumors by using compounds with pro-oxidant activity is developing as a new chemopreventive therapy in cancer.^76^ Exogenous antioxidants not only indiscriminately block indispensable physiological redox-mediated cellular functions; these can also prevent cancer cells from oxidation-induced death.^77^ Indeed, antioxidants such N-acetylcysteine (NAC) or vitamin E (vitE) accelerate tumor progression in mouse models of B-RAF– and K-RAS–induced lung cancer by inactivating p53.^78^ NAC or vitE also potentiates disease progression in melanoma patients by promoting metastasis dependent on NADPH-generating folate pathway^79^ or activation of small guanosine triphosphate RHOA.^80^ However, studies evaluating antioxidants in cancer initiation, particularly in CAC, are scarce.

**Table 1:** Summary of Clinical Studies Using Antioxidants in IBD Patients

Trial characteristic	Population	Subjects	Intervention; duration	Concomitant therapy	Outcome summary	Conclusion	Reference, year
Randomized, placebo-controlled pilot study	UC patients; age: 18–70 y	n =37	Oral NAC (0.8 g/day); 4 weeks	Mesalamine	Clinical remission rate (MTWSI ≤ 2) 63% in treated vs 50% in placebo; clinical response (MTWSI ≥ 2) 66% in treated vs 44% in placebo; reduced interleukin 8 and MCP-1 level; no adverse effect	NAC as combination therapy with mesalamine resulted in clinical improvement in UC patients	87, 2008
Randomized pilot study	UC patients	n=42	Intravenous PC-SOD (40 or 80 mg/day); 4 weeks	Immunosuppressants (azathioprine, mercaptopurine) and/or anti-UC agents (mesalazine, salazosulfapyridine)	Decreased Ulcerative Colitis-Disease Activity Index (UC-DAI) in both 40 mg and 80 mg groups; no severe side effects with any of the doses	PC-SOD improved UC more rapidly than previously existing drugs	58, 2008
Randomized double-blind placebo-controlled	Mild to moderate UC patients; age: 18–75 y	n=121	Oral tablets of PLC (ST 261; 1 g or 2 g/day); 4 weeks	Aminosalicylates or thiopurine	Clinical/endoscopic response in 75% of patients with 1 g/day and 69% in patients with 2 g/day; remission rates were 55%, 49%, and 35% in PLC (1 g/day), PLC (2 g/day), and placebo groups, respectively.	PLC could be potent treatment modality for mild to moderate UC patients	89, 2011
Open-label, proof-of-concept pilot study	Mild to moderate IBD patients; age: 16–80 y	N=14	Oral PLC (ST 261; 2 g/day); 4 weeks	Aminosalicylates, mercaptopurine, or azathioprine	Reduction of Disease Activity Index (DAI) in both UC and CD patients; improvement in Histological Index (HI); no adverse effects	PLC improved endoscopic and histologic activity of mild to moderate UC	88, 2012
Case-control study	IBD patients; age: 15–34 y	n=219; 111 (UC) + 128 (CD)	VitC from food source, calculated from FFQ collected; 5 y	None	Low risk of UC development with vitC intake	Intake of vitC was negatively associated to UC risk	96, 2005
Randomized double-blind placebo-controlled	CD patients; age: 38.3 ± 2.9 y (treated); 36.5 ± 1.7 y (placebo)	n=57	Oral vitC (1000 mg) and vitE (800 IU) daily; 4 weeks	None	Reduction in oxidant burden (measured by breath pentane and ethane output, plasma lipid peroxides, and F2-isoprostane; no change in disease activity	Significant reduction in oxidant burden, but disease activity remained stable in vitC-treated group	93, 2003
Randomized double-blind placebo-controlled	UC patients; age: 20–45 y	n=150	Oral vitA (25,000 IU/day); 2 mo	Mesalamine	Decreased DAI and higher clinical response and mucosal healing in vitA group	VitA had positive clinical and endoscopic effects in UC patients	129, 2018
Open-label study	Mild and moderately active UC patients; age: 21–55 y	n=15	Enema of α-tocopherol (8000 U/day); 12 weeks	Mesalamine	Decreased average DAI, remission in 64% of patients of treated group	α-tocopherol decreased disease severity in patients with active UC	92, 2008
Randomized double-blind placebo-controlled multicentric	Patients with quiescent UC; age: 13–65 y	n=89	Oral curcumin (1 g twice a day); 6 mo	Sulfasalazine or mesalamine	Improved Clinical Activity Index (CAI) and endoscopic index (EI), and suppression in morbidity associated with UC in curcumin group	Curcumin could be a promising and safe medication for maintaining remission in patients with quiescent UC	130, 2006
Randomized double-blind placebo-controlled	Mild to moderate UC patients; age: 18–70 y	n=70	Oral curcumin (500 mg capsule 3 times a day); 8 weeks	Salicylates and/or immunomodulators and/or corticosteroids	Significant improvement in Clinical Colitis Activity Index, significantly higher score of quality of life, reduced serum hs-CRP and ESR in curcumin group than placebo	Curcumin supplementation along with traditional drug was associated with improved clinical outcome in mild to moderate UC patients	131, 2020
Randomized double-blind placebo-controlled	Mild to moderate UC patients; age: 18 y and older	n=56	Oral curcuminoids nanomicelles (80 mg 3 times a day); 4 weeks	Mesalamine	Decreased SCCAI score in curcuminoid group; reduced frequency of urgent defecation; improved patient’s self-reported well-being	Curcuminoids nanomicelles treatment significantly improved clinical activity of UC patients	132, 2018
Randomized double-blind placebo-controlled	Mild to moderate UC patients; age: 18–70 y	n=50	Oral curcumin capsules (1000 mg capsule twice a day); 4 weeks	Mesalamine	Clinical remission in 53.8% and endoscopic remission in 38% of curcumin group compared with 0% in placebo	Addition of curcumin to drug (mesalamine) therapy was superior in inducing clinical and endoscopic remission in UC patients	133, 2015
Randomized double-blind placebo-controlled pilot study	Patients with mild to moderate distal UC; age: >18 y	n=45	Enema of NCB-02 (standardized curcumin preparation) ie, equivalent to 140 mg curcumin once daily; 8 weeks	Mesalamine	Significantly better response in NCB-02 compared with placebo in terms of clinical response (92.9% vs 50%), clinical remission (71.4% vs 31.3%), and improvement in endoscopic activity (85.7% vs 50%)	NCB-02 enema improved disease activity in patients with mild to moderate distal UC	134, 2014
Randomized double-blind placebo-controlled multicentric	Mild to moderate Crohn’s disease patients; age: 21–65 y	n=30	Theracurmin (a new curcumin derivative with increased absorption rate; 360 mg/day); 12 weeks	Mesalamine (90% of patients), immunomodulators (33.3% of patients), steroids (3.3% of patients), and anti-TNFα (6.7% of patients)	Reduction in clinical disease activity; 40% clinical remission rate and 15% endoscopic remission rate in the Theracurmin group compared with 0% in placebo; better healing of anal lesion with no adverse effect in Theracurmin-treated group	Theracurmin treatment showed significant clinical and endoscopic efficacy with favorable safety profile in mild to moderate Crohn’s disease	135. 2020
Randomized double-blind placebo-controlled pilot study	Mild to moderate UC patients; age: >18 y	n=56	Oral resveratrol capsule (500 mg pure trans-resveratrol/day); 6 weeks	—	Decreased disease activity, increased quality of life, increased serum SOD and TAC, and decreased serum MDA in resveratrol group	Supplementation of resveratrol reduced oxidative damage and improved quality of life and disease activity of UC patients	136, 2016
Randomized double-blind placebo-controlled pilot study	Mild to moderate UC patients; age: >18 y	n=50	Oral resveratrol capsule (500 mg pure trans-resveratrol/day); 6 weeks	—	Reduction in plasma levels of TNFα and hs-CRP; suppression of NF-kB in peripheral blood mononuclear cells, decrease in clinical colitis activity index score and increase in IBDQ-9 in resveratrol group	Supplementation of resveratrol reduced inflammation and improved quality of life and colitis activity of UC patients	137, 2015

ESR, erythrocyte sedimentation rate; FFQ, food frequency questionnaire; hs-CRP, high sensitivity C-reactive protein; IBDQ-9, inflammatory bowel disease questionnaire-9; MCP-1, monocyte chemoattractant protein-1; MDA, malondialdehyde; MTWSI, Modified Truelove-Witts Severity Index; PC-SOD, lecithinized superoxide dismutase; PLC, propionyl-L-carnitine; SCCAIQ, Simple Clinical Colitis Activity Index Questionnaire; SOD, superoxide dismutase; TAC, total antioxidant capacity.

**Table 2:** Summary of Clinical Trials Using Antioxidants in Patients Diagnosed With CRC

Trial characteristic	Population	Subjects	Intervention; duration	Outcome summary	Conclusion	Reference, year
Randomized, double-blind, placebo controlled (ATBC study)	Male smokers of southwestern Finland; age: 50–69 y	n=29,133	Oral alpha-tocopherol (50 mg/day) or beta-carotene (20 mg/day) or combination of both; 5–8 y	CRC incidence was modestly lower but not significant in alpha-tocopherol group (RR = 0.78; 95% CI, 0.55–1.09); beta-carotene had no effect on CRC incidence (RR = 1.05; 95% CI, 0.75–1.47)	No response on CRC incidence in older male smokers	138, 2000
Randomized, double-blind, placebo controlled (ATBC study)	Male smokers of southwestern Finland; age: 50–69 y	N=15,538	Oral alpha-tocopherol (50 mg/day) or beta-carotene (20 mg/day) or combination of both; 6.3 y	Alpha-tocopherol increased the risk of adenoma (RR = 1.66; 95% CI, 1.19–2.32); beta-carotene had no effect on adenoma risk (RR = 0.98; 95% CI, 1.71–1.35)	Negative response; alpha-tocopherol increased the risk of adenoma; however, beta-carotene had no effect on adenoma in older male smokers	139, 1999
Randomized, controlled clinical trial	Patients post-removal of at least 1 colonic adenoma	n=864	Oral beta carotene (25 mg/day) or vitC (1 g/day) and vitE (400 mg/day); 4 y	RR for beta carotene was 1.01 (95% CI, 0.85–1.20) and for vitC and E was 1.08 (95% CI, 0.91–1.29)	No response; neither treatment was effective in prevention of any subtype of polyp irrespective of size and location	114, 1994
Prospective interventional study	Patients previously diagnosed with colorectal adenomas; age: 50–76 y	n= 116	Oral antioxidants and calcium tablet once daily that contains beta-carotene (15 mg), vitC (150 mg), vitE (75 mg), selenium (101 μg), and calcium (1.6 g); 3 y	No difference was detected in growth of adenomas between treated and placebo groups; significantly lower number of patients free of new adenomas in placebo group compared with treated group	No response on polyp growth*;* positive response on protection from developing new adenoma	140, 1998
Randomized, double-blind, placebo-controlled	Patients with history of sporadic colorectal adenoma; age: 30–74 y	n=47	Oral antioxidant micronutrient cocktail delivering vitE (800 mg), beta-carotene (24 mg), vitC (1 g), selenium (200 μg), riboflavin (7.2 mg), niacin (80 mg), zinc (60 mg), and manganese (5 mg) per day; 4 mo	TNF-α decreased by 37% and cystine decreased by 19% in antioxidants treatment group relative to placebo; interleukin 6 and F2-isoprostane levels decreased in antioxidant-treated nonsmokers but increased in smokers	Positive response only in nonsmoker subjects; an antioxidant micronutrient cocktail decreased the level of oxidants and inflammation only in nonsmokers	141, 2010
Randomized, controlled study	Patients with colonic polypectomy; mean age: 59.2 y	n=255	Oral vitamins tablet containing vitC (1 g/day), vitA (30,000 IU/day), and vitE (70 mg/day); ∼5 y	Percentage of recurrence of adenomas was 5.7% in vitamins group compared with 35.9% in untreated group	Positive response; vitamins treatment lowered recurrence rate of colonic adenomas	142, 1993
Randomized, double-blind trial	Patients post-removal of at least 1 colonic adenoma	n=200	Oral vitC (400 mg/day) and vitE (400 mg/day); 2 y	Difference in incidence of polyp recurrence was small in treated group compared with placebo (RR = 0.86; 95% CI)	Positive response (small effect); small reduction in rate of polyp recurrence with vitamin supplement	143, 1988
Randomized, double-blind, placebo-controlled	Patients with advanced colonic adenocarcinoma	n=100	Oral vitC (10 g/day) as capsule; 2 y	No benefit with high-dose vitC either as disease progression or survival compared with placebo	No response on either overall survival or progression of advanced CRC	144, 1985
Pilot study	Patients with terminal cancer including colon cancer; age: 32–93 y	n=100 vitC treated and 1000 control subjects	VitC; 10 g/day IV for 10 days followed by 10 g/day oral; ∼ 210 days	Survival was about 4.2 times greater in treated group (∼210 days) compared with control group (∼50 days)	Positive response on overall survival; treatment with vitC increased survival time by about 3 times in terminal cancer patients	145, 1976
Randomized, double-blind, placebo-controlled	Patients with large bowel adenoma/polyposis coli; age: 20–63 y	n=36	Oral vitC (3 g/day); ∼2 y	Reduction in both number and area of rectal polyps in vitC group at 9 months of follow-up	Positive response (temporary, only at 9 months of follow-up) on reduction of polyp growth and turnover	112, 1982
Phase 1 open-label, single-center, dose escalation, and speed-expansion study	Metastatic colorectal cancer (mCRC) or gastric cancer (mGC); age: 18–75 y	n=36	VitC infusion in dose escalation (0.2–1.5 g/kg) and in speed expansion study (1.5 g/kg) once daily for 3 days in 14-day cycle in combination with mFOLFOX6 or FOLFIRI; 12 cycles	Maximum tolerated dose of vitC not achieved; recommended phase 2 dose of vitC at 1.5 g/kg/day was established; response rate was 58.3%, and disease control rate was 95.8% in treated group	Positive response as combination therapy; favorable safety profile and potential clinical efficacy were observed with combined treatment of vitC and mFOLFOX6/FOLFIRI	146, 2019
Randomized, placebo-controlled trial, Selenium and vitE Cancer Prevention Trial (SELECT)	SELECT participants who underwent lower endoscopy; age: ≥50 y (African American), ≥55 y (all other men)	N=8094	Oral selenium (200 μg/day) and vitE (400 IU/day); 7–12 y	RR for adenoma occurrence in selenium group was 0.96 (95% CI, 0.90–1.02) and in vitE group was 1.03 (95% CI, 0.96–1.10) compared with placebo	No response on colorectal adenoma occurrence	147, 2017
Randomized, placebo-controlled trial	Patients post-removal of at least 1 colorectal adenoma; age: 40–80 y	n=1621	Selenium (200 μg/day) as selenized yeast in combination with celecoxib (400 mg daily); ∼33 mo	RR of adenoma in selenium group was 1.03 (95% CI, 0.91–1.16) compared with placebo; adenoma recurrence in patients with baseline advanced adenomas was reduced by 18% with selenium	No response on colorectal adenoma formation but showed only modest benefit on adenoma recurrence	148, 2016
Randomized, placebo-controlled trial	Patients with confirmed recent histories of nonmelanoma skin cancer; age: <80 y	n=1312	Selenium (200 μg/day) as selenized yeast; 7.9 y	Suggestive but nonsignificant decrease in risk associated with selenium on prevalent adenomas (odds ratio = 0.67; 95% CI, 0.43–1.05); significant reduced risk was observed in subjects with lowest baseline selenium and current smokers	Positive response only in subjects with low baseline selenium or smoking habit	149, 2006
Randomized double-blind placebo-controlled	Post-polypectomy (colonic) patients; age: 29–83 y	n=411	One tablet daily composed of 200 μg selenium, 30 mg zinc, 2 mg vitA, 180 mg vitC, and 30 mg vitE; 5 y	A 39% reduction in risk of adenoma recurrence with intervention compared with placebo; similar risk reduction was also observed in small tubular and advanced recurrent adenomas	Positive response on adenoma recurrence	150, 2013
Randomized, placebo-controlled, prospective trial	Patients post-surgical resection of colon or rectal adenocarcinoma; age: 50–75 y	n=24	Oral zinc capsules (70 mg/day) in combination with capecitabine or capecitabine with oxaliplatin/5-fluorouracil; 16 weeks	No change in plasma level of vitC, vitE, MDA, or 8-isoprostane but increased SOD activity in zinc-treated group compared with placebo	No response on lipid peroxidation markers but improved SOD activity in zinc-treated group	151, 2016
Randomized, double-blind, placebo-controlled	Patients with familial adenomatous polyposis; age: 18–85 y	n=44	Oral curcumin (3000 mg/day); 12 mo	No significant difference in mean polyp number or size was observed between curcumin and placebo-treated groups	No response on polyp number and size in FAP patients	152, 2018
Randomized, open-labelled, controlled trial	Patients with metastatic colorectal cancer; age: >18 y	n=28	Oral curcumin C3 complex/d (2 g/day) in combination with FOLFOX; ∼24 weeks	Daily oral supplementation of curcumin to FOLFOX chemotherapy was safe and tolerable; no significant difference between arms for quality of life or neurotoxicity	No response on quality of life, but curcumin could be safe and tolerable adjunct to FOLFOX chemotherapy in patients with metastatic CRC	153, 2019
Single-center prospective randomized open-labelled	Patients with colonic polypectomy; age: 19–85 y	n=176	Oral GTE as tablet (0.9 g/day) equivalent to 0.6 g/day of catechin or 0.2 g/day of EGCG; 12 mo	Decreased incidence of metachronous adenoma and number of relapsed adenomas in GTE group	Positive response on metachronous colorectal adenomas	154, 2018
Pilot study	Patients with colonic polypectomy; age: 20–80 y	n=136	Oral GTE as tablet (1.5 g/day); 12 mo	Decreased incidence of metachronous adenoma and smaller size of relapsed adenomas in GTE group	Positive response on metachronous colorectal adenoma	155, 2008
Prospective cohort study	Patients with resected colon cancer or polypectomy; age: median age 74 and 77 for treated and control groups, respectively	n=87	Oral flavonoid mixture consists of apigenin (20 mg) and epigallocathechin-gallat (20 mg) daily; 4 y	Recurrence rate for neoplasia was 7% in treated group compared with 47% in control group	Positive response with long-term treatment on recurrence rate of colon neoplasia	156, 2008
Randomized, placebo-controlled trial	Patients with previous adenomatous colonic polyps	n=64	Oral NAC (800 mg/day) as capsule; 12 weeks	Proliferative index of colonic epithelial cells was reduced in NAC group in comparison with placebo group	Positive response on reducing colonic epithelium hyperproliferation; could be a chemopreventive agent in human colon cancer	157, 1999
Randomized and controlled	Patients with gastrointestinal cancer undergoing major abdominal surgery	n=33	NAC (1200 mg/day) through parenteral nutrition starting from 2 days before surgery until fifth post-surgery day; 7 days	Reduced plasma MDA but higher ratio of reduced to oxidized glutathione in NAC group; no change in plasma level of vitA, vitC, or vitE but reduction in urinary nitrate level with NAC treatment	Positive response on reducing oxidant and improving antioxidant parameters in cancer patients undergoing major abdominal surgery	158, 2015

ATBC, Alpha-Tocopherol, Beta-Carotene Cancer Prevention Study; CI, confidence interval; EGCG, (-)-epigallocatechin gallate; GTE, green tea extract; MDA, malondialdehyde; RR, relative risk.

## Testing Exogenous Antioxidants in IBD

Investigators have examined the protective role of synthetic compounds such as inhibitors of pro-oxidant enzymes and precursor of endogenous antioxidants in IBD. Among several drugs, inhibitors of angiotensin II type 1 (AT1) and hydroxymethylglutaryl coenzyme A reductase are reported to have both antioxidant and anti-inflammatory activities. AT1 increases mitochondrial production of O_2_^•-^ and H_2_O_2_ through NADPH oxidase and inflammation through nuclear factor kappa B. Accordingly, the AT1 antagonist telmisartan was protective in DSS-induced colitis.^81^ Hydroxymethylglutaryl coenzyme A inhibitors such as simvastatin, rosuvastatin, and pravastatin are primarily lipid-lowering drugs, but they ameliorate disease severity in colitis models by reducing inflammation and inducing endogenous antioxidants such as SOD and glutathione.^82^^,^^83^

As discussed earlier, glutathione is depleted in IBD patients. Administration of NAC, a synthetic precursor of glutathione, in colitis models ameliorated colitis severity,^84^, ^85^, ^86^ but not all studies are in agreement.^3^ NAC treatment in UC patients resulted in a significant improvement in clinical features and a reduction in serum proinflammatory cytokines.^87^ Similarly, restoring colonic SOD level by administering lecithinized SOD, a synthetic SOD mimic, improved colitis severity in preclinical and human studies.^57^^,^^58^ Other synthetic antioxidants such as propionyl-L-carnitine, an ester derivative of L-carnitine, improved disease in mild to moderate UC patients.^88^^,^^89^

Natural exogenous antioxidants have also been examined in IBD. VitE is a lipid-soluble vitamin primarily involved in protecting cell membrane from oxidative damage. Supplementation of vitE in preclinical models of colitis ameliorated colitis severity.^90^^,^^91^ However, results from clinical studies are inconclusive.^92^^,^^93^ Vitamin C (vitC) is a water-soluble vitamin that acts as a potent antioxidant because of its ability to donate electrons. Low or high doses of vitC reduce inflammation in animal models^94^^,^^95^; however, not all studies are in agreement.^3^ Clinical studies using vitC in IBD patients are also inconsistent.^93^^,^^96^

Altogether, the use of exogenous antioxidants to treat IBD needs further work because many studies are preliminary especially in clinical trials, and there are many conflicting findings (Table 1).

## Testing Exogenous Antioxidants in CAC

Despite some promising albeit conflicting results with exogenous antioxidants in treating IBD, it is possible that many antioxidants have little to no anti-inflammatory activity and thus may not be useful in treating IBD. On the other hand, exogenous antioxidants could be used to protect from CAC by reducing oxidative DNA damage. However, only a few studies have evaluated antioxidants in preclinical models of CAC.

Long-term administration of NAC reduces oxidative damage (nitrotyrosine and 8-oxoG) in colonic mucosa and protects from CAC development.^3^^,^^97^^,^^98^ In addition to its role in cellular redox signaling, peroxynitrite, which is a coupling product of nitric oxide and superoxide, can oxidize DNA and produce DNA lesions. Accordingly, L-NIL, an inducible nitric oxide synthase inhibitor, reduced 8-oxoG levels and colonic polyps in multiple mouse models of CAC, despite having no significant effect on mucosal inflammation.^3^ In agreement with this study, a derivative of L-NIL (SC-51) reduced inducible nitric oxide synthase and COX-2 activities and lessened the incidence of AOM-induced colonic aberrant crypt foci in rats.^99^ These findings suggest that limiting nitrosative DNA damage might curb CAC development. Other synthetic compounds such as GL-V9, a flavonoid derivative with strong antioxidant and anti-inflammatory activities, protect against tumorigenesis in a CAC model through NLRP3 inflammasome degradation.^100^ Statins were found to reduce CAC in IBD patients in one study^101^ but not in another study.^102^

Among natural antioxidants, vitC reduces oxidative DNA damage by neutralizing mutagenic ROS and RNI^3^^,^^103^ and protects from inflammation-associated tumorigenesis in different animal models of CAC.^3^ Paradoxically, a pro-oxidant role for vitC has also been reported at high doses or in presence of transition metals.^104^ High doses of vitC induce cytotoxicity in cancer cells,^105^ and it was thus evaluated as a therapeutic agent in CRC patients. However, the results are inconsistent^106^ (Table 2). Although studies on therapeutic evaluation of curcumin in CAC are limited, some preclinical studies have found a reduction in colonic tumor burden in CAC models.^107^^,^^108^ The effects of resveratrol on CAC have been evaluated in one study that reported a reduction in tumor incidence in AOM/DSS model.^109^^,^^110^

Although there have been numerous trials investigating the effects of antioxidants on disease pathology in IBD patients with mixed results (Table 1), few have used CAC as an endpoint. In light of the findings in preclinical models, serious consideration should be taken to test the role of antioxidants to prevent CAC in IBD patients.

## Concluding Thoughts

Several translational studies have shown that antioxidants are effective at reducing both an overt oxidative environment and oxidative DNA lesions in the intestine and other tissues.^3^^,^^93^^,^^95^^,^^103^ For years these studies have supported the belief that antioxidants can protect DNA from oxidative damage that could precipitate cancer. However, clinical studies that have tested this hypothesis have not reached consistent results.^111^, ^112^, ^113^, ^114^, ^115^ One factor that could explain these contradictory results is that antioxidants have been promoted and tested as the panacea for all cancers. Although an excess in oxidative molecules could theoretically induce tumor initiating DNA lesions in any cell, susceptibility to oxidative DNA damage is expected to vary widely in different tissues.^78^^,^^116^^,^^117^ Differences in cell proliferation, gene expression, and the cell’s oxidative environment are expected to influence the probability of acquiring oxidative DNA mutations. For example, because oxidative DNA lesions that occur during S-phase of the cell cycle are more likely to result in mutations (Figure 2), highly proliferative tissues such as the intestine are more susceptible to acquire tumor-initiating mutations in oxidative environments. In addition, some tissues such as the intestine are in close contact with microbes and as a result are in a harsh oxidative environment produced by immune cells to keep microbes in check. It is therefore expected that oxidative DNA lesions only promote certain types of cancers.^116^ Indeed, an analysis of mutational signatures in more than 40 different cancers found that most of colorectal and stomach adenocarcinomas have a ROS mutational signature, whereas other cancers do not.^116^

The path of genetic mutations that are required for cellular transformation will be different in different tissues and cells. This depends on a number of factors such as the cellular environment and the type of cell being transformed. In the case for CRC and CAC, the cell type that is transformed is similar, but the environments where the cancers arise are different, which might explain the different genetic mutations associated with each of these cancers. For example, whereas mutations that affect the Wnt pathway occur in 85% of sporadic CRCs^118^ and are considered to be the first step that leads to CRC initiation, CAC tumors first acquire mutations in p53, followed by KRAS mutations.^119^ p53 is a transcription factor that controls the DNA damage response by inducing cell cycle arrest and apoptosis.^120^ However, p53 is also involved in other cellular processes such as the antioxidant response, and its down-regulation results in increased DNA oxidation and mutation rates in lymphoma models.^120^^,^^121^ Hence, it is tempting to speculate that inactivation of genes that regulate the antioxidant response is more important for the development of CAC than CRC possibly because of the high oxidative environment of the inflamed gut. This notion is supported by findings that antioxidants only reduced tumorigenesis in CAC models but not in a familial model of CRC (ie, MMR-deficient Lynch syndrome).^3^ This result could be explained by the fact that most mutations in MMR-deficient cells are due to replication errors and spontaneous cytidine deamination, with only 20% of mutations potentially attributed to oxidative DNA lesions.^122^ Hence, most mutations that appear in MMR-deficient cells cannot be prevented with antioxidant treatment. In contrast, antioxidants reduced tumorigenesis by 50% in all CAC models tested,^3^ suggesting that a larger fraction of genetic lesions in inflamed colons is a consequence of oxidative DNA damage. This argument might provide an explanation for the conflicting results in clinical trials that tested antioxidants in CRC and suggests that clinical trials using antioxidants should stratify patients according to genetic susceptibility to acquire oxidative DNA lesions.

Importantly, because ROS are required to maintain homeostasis in the intestinal epithelium, antioxidants should be administered with precaution. Molecules such as O_2_^•-^ and H_2_O_2_ have both proliferative and antiproliferative effects and can regulate cell differentiation, intestinal repair, and antimicrobial defense.^8^, ^9^, ^10^, ^11^, ^12^ Therefore, completely shutting off Redox signaling could potentially disrupt homeostasis and cause disease. Indeed, NOX1, NOX2, and DUOX2 deficiencies have been associated with a higher risk of developing pediatric^123^ and very early onset IBD.^124^, ^125^, ^126^ Furthermore, patients suffering from chronic granulomatous disease, a rare disorder characterized by deficiency in phagocytic NOX function, have a high risk to develop IBD,^127^^,^^128^ suggesting that defective O_2_^•-^ production can lead to IBD and therefore antioxidant doses should be carefully adjusted for these patients.

In conclusion, both in vitro and in vivo studies suggest the potential role of ROS and RNI in the pathophysiology of IBD and CAC. Epidemiologic studies show altered levels and activity of endogenous antioxidants in IBD and CAC patients. However, further studies are required to confirm their association with disease pathophysiology. There are some promising results from preclinical studies that the use of exogenous compounds (natural or synthetic) with antioxidant activity prevents oxidative DNA damage and CAC. However, this treatment strategy needs to be confirmed in the clinic.
